# Ictal asystole: a case presentation

**DOI:** 10.1186/s12883-018-1105-5

**Published:** 2018-07-21

**Authors:** Nirmeen Kishk, Amani Nawito, Ahmed El-Damaty, Amany Ragab

**Affiliations:** 1 Neurology Department, Faculty of Medicine, Kasr Alainy Hospital, Cairo University, Cairo, Egypt; 2 Clinical Neurophysiology Unit, Faculty of Medicine, Kasr Alainy Hospital, Cairo University, Cairo, Egypt; 3 Cardiovascular Department, Faculty of Medicine, Kasr Alainy Hospital, Cairo University, Cairo, Egypt

**Keywords:** Ictal asystole, Epilepsy, Cardiac arrhythmia, Pacemaker, Simultaneous ECG and EEG

## Abstract

**Background:**

Epileptic seizures can lead to cardiac arrhythmias. The arrhythmias may be in the form of tachycardia, bradycardia or asystole. Ictal bradycardia and asystole can lead to sudden unexpected death.

**Case presentation:**

A case report of a 40-year-old male with complex partial temporal lobe epilepsy. He has coincident attacks of fall and pallor. The patient underwent simultaneous electrocardiogram (ECG) and video electroencephalogram (EEG) monitoring. The slow activity in EEG coincide with the appearance of bradycardia in ECG then cardiac asystole which clinically correspond to the patient syncope. After insertion of a cardiac pacemaker, only complex partial attacks develop with a marked reduction in frequency and no more fall attacks.

**Conclusion:**

Epileptic seizures can present with cardiac arrhythmias, with ictal asystole leading to sudden unexpected death. Simultaneous EEG and ECG are essential for the diagnosis. A cardiac pacemaker can be lifesaving for patients with ictal arrhythmias.

## Background

Epileptic seizures can affect the heart rate leading to arrhythmia [1]. The most common arrhythmia associated with epilepsy is ictal tachycardia (80–100% of all seizures) [2]. Ictal bradycardia occurs in fewer than 6% of seizures. This slowing of the heart rate may be severe enough to cause ictal asystole. Ictal asystole defined as the absence of ventricular complexes for more than 4 s, accompanied by electrographic seizure onset [3].

Ictal asystole is found in 0.27–0.4% of patients undergoing video-EEG. Clinically, a loss of epileptic activity occurs due to brain anoxia along with a loss of muscle tone and consciousness [4, 5]. Approximately 80% of cases are associated with temporal lobe epilepsy while 20% of cases occur with extratemporal lobe seizures [6, 7].

Ictal bradycardia and ictal asystole may lead to sudden unexpected death in epilepsy patients (SUDEP) [8, 9].

## Case presentation

The patient is a 40- years- old right-handed Egyptian male accountant with a negative perinatal history, no family history of epilepsy, no consanguineous marriage, and no medical comorbidities.

At the age of 23, after graduating from university, his father noticed recurrent nocturnal attacks in the form of right-sided head and neck deviation with tonic movements in both the upper and lower limbs. The episodes lasted for approximately 30–40 s and recurred 2–3 times on the same night with no tongue biting or urinary incontinence.

Conventional EEG showed left temporal epileptiform discharge.

Improvement was observed with carbamazepine (400 mg/day), and the patient became seizure-free for 1 year.

At the age of 32, he started to develop recurrent attacks with the following characteristics: A prodromal sense of dizziness followed by loss of contact with the environment, automatism, and stereotyped motor movement in both the upper and lower limbs (marching movements). He became pale and then experienced a loss of tone, causing him to fall to the ground, lasting 40–60 s. No tongue biting or urinary incontinence were reported, but sometimes self-injuries occurred. Then the patient regained consciousness after approximately 15 min of confusion. Each time he asked, “What day is it today?” and “What time is it?” This episode was followed by a sense of fatigue.

These attacks did not occur out of sleep and recurred every 1–2 weeks.

Neurological Examination, conventional EEG, and brain magnetic resonance imaging (MRI) were normal.

The patient was prescribed levetiracetam 2000 mg /day, oxcarbazepine 500 mg/day, clonazepam 0.5 mg /day, and lacosamide 150 mg per day with a partial reduction in the frequency of the attacks.

When patient attended our clinic, he was monitored to ensure that he was compliant with his medications, and the doses were adjusted according to his body weight. We increased the doses and gave him 4 anti-epileptic drugs (AEDs) (Levetiracetam 3000 mg, oxcarbazepine 900 mg, lacosamide 200 mg, clonazepam 0.5 mg/day).

The frequency of events (dizziness, loss of contact, motor automatism) was reduced. However, the attacks of pallor and falling still recurred once per month. Sometimes the attacks were related to psychological or mental stress. Thus, the patient was referred for overnight video-EEG in our unit.

**Fig. 1:**
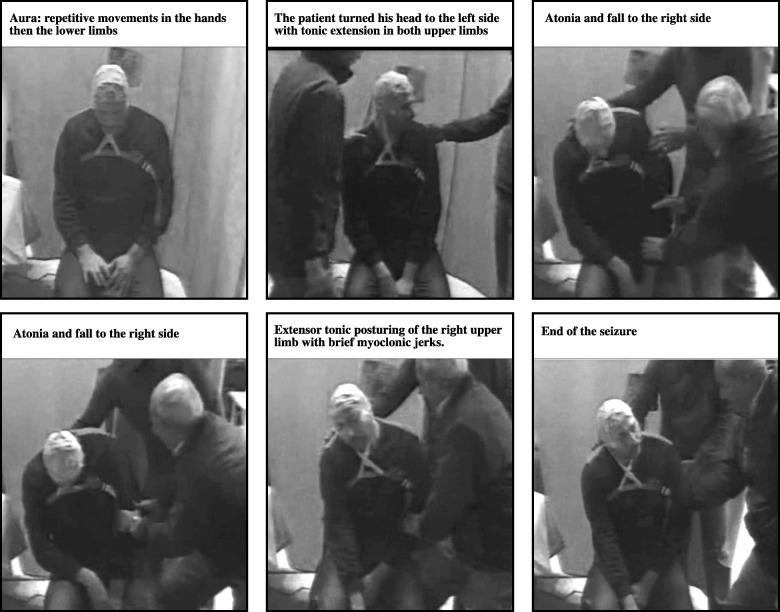
Screenshots from the video- EEG Results

The video- EEG recording was performed overnight using a Nihon Kohden Neurofax 9200 EEG apparatus (Tokyo, Japan). The electrodes were applied according to the 10–20 electrode placement system, in addition to T1 and T2 electrodes. An additional ECG channel was included. Three minutes of hyperventilation and photic stimulation were performed as provocative procedures (Fig. 1).

**Fig. 2:**
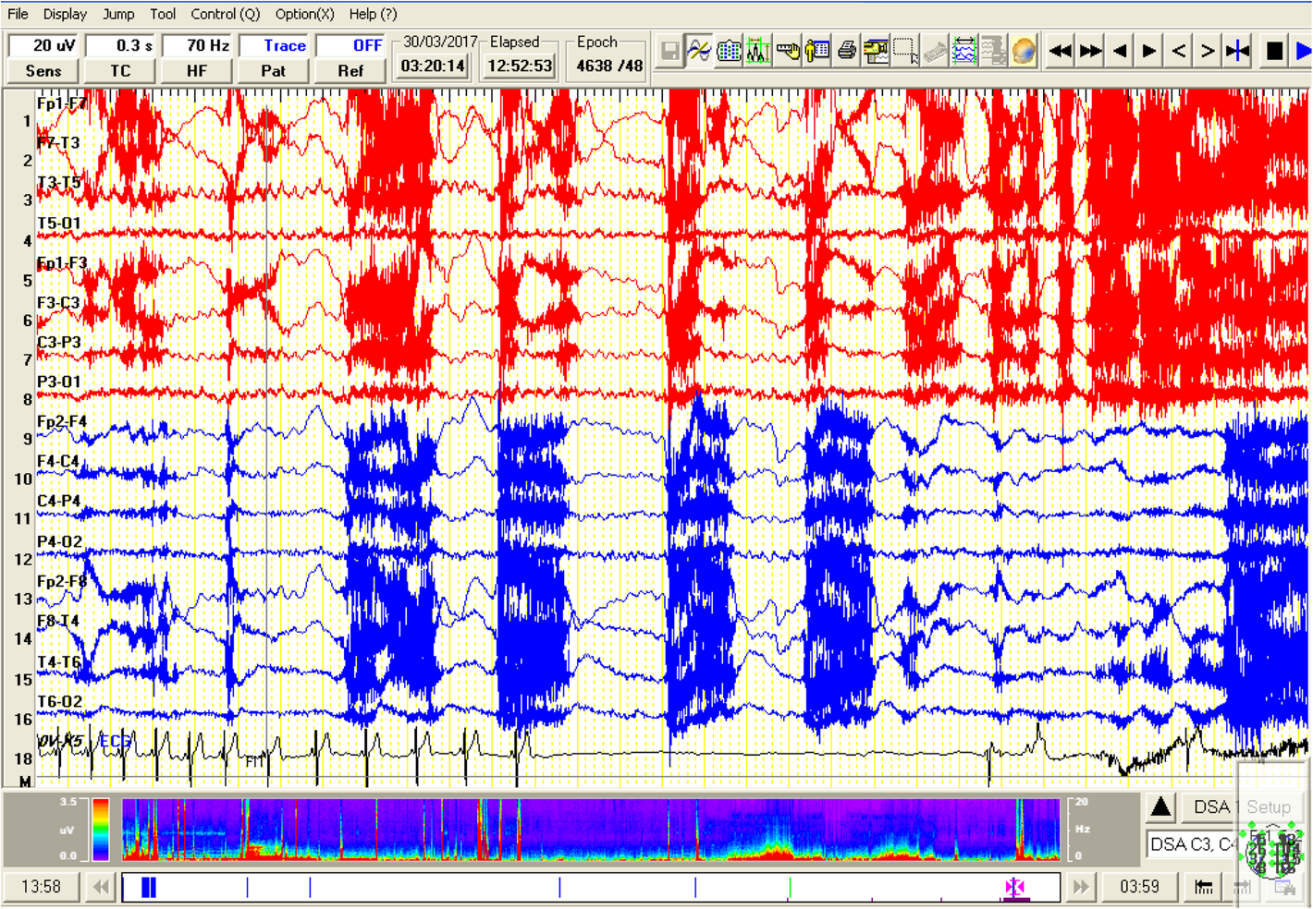
A compressed EEG segment (30 s) showing bilateral temporal ictal EEG discharges more on the left side followed by the period of asystole

The patient was awake when his father indicated that the usual seizure had started. The patient had lost contact and began experiencing motor automatism in the form of repetitive movements of the right hand, then the left hand, and finally, the lower limbs. The EEG showed rhythmic alpha frequencies over the left temporal region evolving into theta frequencies that involved both hemispheres. Then, the patient turned his head to the left side and displayed tonic movement in both upper limbs. The simultaneous EEG revealed bilateral, rhythmic temporal theta frequencies. The ECG channel showed 5 s of bradycardia then a 10- s asystole (Fig. 2). Approximately 7 s after the onset of asystole, the patient had atonia and fell to the right side (syncope). The EEG indicated diffuse slowing with amplitude suppression. Next, the patient showed tonic extensor posturing of the right upper limb and brief myoclonic jerks. Here, the EEG was masked by movement and electromyogram (EMG) artifacts. Meanwhile, the ECG revealed bradycardia and then a regular rhythm.

After video EEG ictal recording we decided to stop lacosamide 200 mg to avoid the aggravation of the cardiac arrhythmia. In the same week, the patient was referred for permanent cardiac pacemaker implantation and advised to continue his prescribed antiepileptic medications.

After implantation of a dual chamber pacemaker, only complex partial attacks occurred and did so with a lower frequency. No additional falling attacks followed.

## Discussion

A complex relationship exists between seizures and the heart.

Epileptic activity originating in the amygdala, insular cortex, cingulate gyrus, frontopolar region, and frontotemporal region can produce a broad range of cardiac abnormalities, including supraventricular tachycardia, sinus tachycardia, sinus bradycardia, sinus arrest, atrioventricular block, and asystole [4].

Intraoperative stimulation of the human insular cortex reveals that right insular stimulation leads to tachycardia and pressor responses, while left insular stimulation leads to bradycardia and depressor responses [10].

Ictal bradycardia is observed in patients with a long history of epilepsy, especially those with refractory seizures. This may occur because repeated seizures or antiepileptic drugs use impairs the neurocardiac regulatory system [11].

Ictal asystole should be suspected if the typical seizure semiology is associated with syncopal episodes [12, 13].

Simultaneous EEG and ECG recordings are the only methods to differentiate between primary cerebrogenic and cardiogenic causes of arrhythmias. In primary cerebrogenic bradyarrhythmia, EEG seizure activity precedes the onset of bradyarrhythmia. Also, a 24-h Holter ECG is essential to exclude intrinsic cardiac disease, which may be a predisposing factor for ictal asystole [10].

This case is a diagnostic challenge and difficult to be diagnosed unless in a highly specialized integrated epilepsy unit with a rapid and easy referral and effective communication between departments.

## Conclusion

Epileptic seizures can present with cardiac arrhythmias and ictal asystole, which may lead to sudden unexpected death. Simultaneous EEG and ECG recordings are essential for diagnosis. A cardiac pacemaker can be lifesaving for patients with ictal arrhythmias.

## Abbreviations

AEDs: Anti-epileptic drugs
ECG: Electocardiogram
EEG: Electroencephalogram
EMG: Electromyogram
MRI: Magnetic resonance imaging
SUDEP: Sudden unexpected death in epilepsy patients
